# Strong natural selection during plant restoration favors an unexpected suite of plant traits

**DOI:** 10.1111/eva.12038

**Published:** 2013-01-03

**Authors:** Sarah M Kulpa, Elizabeth A Leger

**Affiliations:** Department of Natural Resources and Environmental Science, University of NevadaReno, NV, USA

**Keywords:** *Elymus elymoides*, functional traits, natural selection, phenology, plant size, seed size, selection differential

## Abstract

Restoration is an opportunity to study natural selection: One can measure the distribution of traits in source propagules used to found populations, compare this with the distribution of traits in successful recruits, and determine the strength and direction of selection on potentially adaptive traits. We investigated whether natural selection influenced seedling establishment during postfire restoration in the Great Basin, an area where large-scale restoration occurs with a few widely available cultivars planted over a large range of environmental conditions. We collected seeds from established plants of the perennial grass *Elymus elymoides* ssp. *californicus* (squirreltail) at two restoration sites and compared the distribution of phenotypic traits of surviving plants with the original pool of restoration seeds. Seeds were planted in common gardens for two generations. Plants grown from seeds that established in the field were a nonrandom subset of the original seeds, with directional selection consistently favoring a correlated suite of traits in both field sites: small plant and seed size, and earlier flowering phenology. These results demonstrate that natural selection can affect restoration establishment in strong and predictable ways and that adaptive traits in these sites were opposite of the current criteria used for selection of restoration material in this system.

## Introduction

Natural selection affects the distribution and abundance of phenotypes in the wild (Endler 1986; Kingsolver et al. 2001; Geber and Griffen 2003). Traits with strong fitness effects and high heritability are expected to respond to selection, leading to adaptation to local environments (e.g., Turesson 1922; Clausen et al. 1941; Hiesey et al. 1942; Leimu and Fischer 2008). Understanding which traits increase fitness in particular environments and at specific life-history stages is a focus of basic evolutionary and ecological research, though it can often be impractical or impossible to perform the experiments necessary to observe the relationship between phenotype and fitness in wild populations. Ecological restoration can provide the conditions under which such experiments can be conducted, and often over large spatial and temporal scales. Just as restoration ecology can serve as an acid test for ecological theory by testing our understanding of community assembly and ecosystem function (Bradshaw 1987), restoration can also test predictions about the consistency of natural selection and the importance of specific traits for increasing organism fitness in field settings.

Plant restoration is an expanding enterprise on degraded and semidegraged land worldwide, as humans attempt to reclaim land after disturbances such as mining, agriculture and plantations, fire, and species invasion alter native communities (Hobbs and Harris 2001; Lamb et al. 2005; Cramer et al. 2008; Davies et al. 2011). Seed provenance is considered a key for restoration success (Lesica and Allendorf 1999; Hufford and Mazer 2003; McKay et al. 2005). Selecting appropriate propagules for restoration can increase plant establishment by ensuring the best match between adaptive traits and environments, and locally collected seeds can outperform nonlocal seeds in restoration (e.g., Humphrey and Schupp 2002; Bischoff et al. 2006; Rice and Knapp 2008; Leimu and Fischer 2008). However, as ecosystems are altered by invasive species, climate change, and modified disturbance regimes, identifying restoration material that performs well under specific conditions may lead to more effective restoration than selection criteria based solely on local provenance (Wilkinson 2001; Rice and Emery 2003; McGill et al. 2006; Millar et al. 2007; Broadhurst et al. 2008; Funk et al. 2008; Leger 2008; Rowe and Leger 2011).

Methods designed to quantify response to natural selection in wild populations (Arnold and Wade 1984; Lande and Arnold 1983; Falconer and Mackay 1996) can help determine if traits or combinations of traits are optimal for a given location in the context of ecological restoration or management. For example, fisheries biologists have measured the strength of selection on particular traits during reintroductions of endangered Atlantic salmon. They have quantified how adaptive traits differ across environments and determined which traits of released fry deviated from local optima (Hendry et al. 2003; Bailey and Kinnison 2010). Differences between local selective optima and the trait distributions of restoration propagules represent inefficiency in management, and if these suites of traits are sufficiently different, mismatch could doom restorations to fail before they begin (Ashley et al. 2003; McKay et al. 2005). Beyond improvements to restoration and management, studies of evolutionary responses during restoration could be useful for studying constraints to evolution (Antonovics 1976; Blows and Hoffmann 2005) or for identifying candidate genes for specific traits of interest (Hoffmann and Willi 2008). Although conceptually straightforward, observations of natural selection during the course of restoration have not been conducted in plant communities.

**Figure 1:**
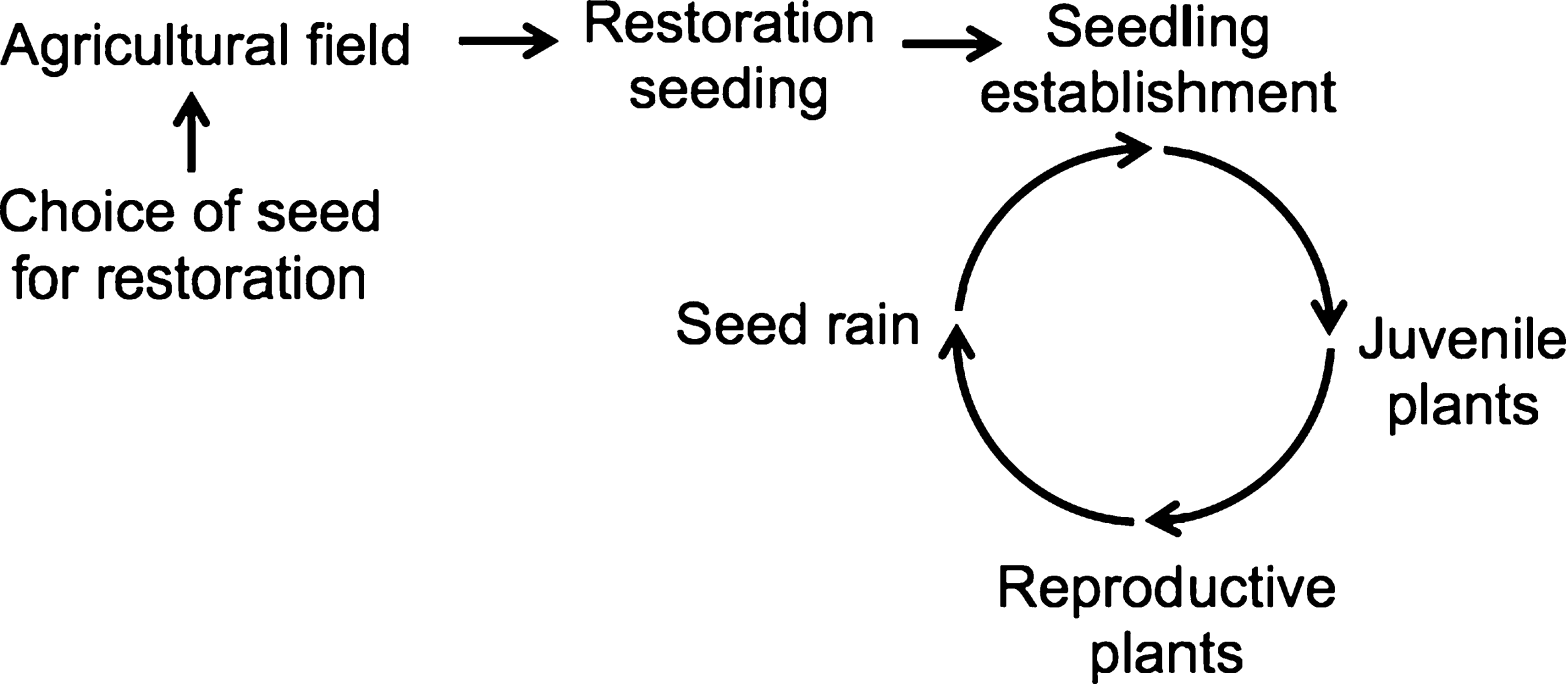
Timeline of restoration and plant establishment, depicting events where artificial or natural selection may be important. When choosing seed for restoration, plant size, seed size, early emergence, and seed production in an agricultural field are among the desired characteristics, and yield is likely to be under selection in agricultural fields. After restoration seeding, selection is imposed by conditions at the restoration site, which may be very different from those in the agricultural field. During the timeframe of this experiment, seed was collected from reproductive plants established during the restoration seeding, and thus, selection measured here likely occurred during the seedling establishment and juvenile phases.

Here, we focus on whether an evolutionary response to natural selection can be detected in the course of a restoration project, focusing on postfire restoration in the Great Basin. In the western USA, restoration occurs across vast acres of degraded private and public lands where annual grass invasion and increased fire frequencies have altered native ecosystems and intervention, often through restoration using native or introduced cultivars, is necessary to restore invaded landscapes back to complex, diverse systems (Young and Evans 1978; D'Antonio and Vitousek 1992; Knapp 1996; USDI BLM 2007; Chambers et al. 2007). Cultivars used for restoration vary in development history and can be composed of collections from one to multiple populations. Further, cultivars may have experienced ‘natural track’ selection (selection of a particular population, but no further manipulations) or may be manipulated releases that have experienced intentional selection and breeding (Jones 2003). In either case, specific traits, such as plant and seed size, seed production, and phenology, are identified as criteria for choosing specific populations to increase for restoration (Gibbs and Young 1989; Jones et al. 2004a,b; Fig. 1). Emerging early can provide individuals with a competitive advantage in resource acquisition over later emerging plants (Cook 1980; Verdú and Traveset 2005; Benard and Toft 2007). Seedlings from larger seeds have been shown to have a higher probability of emergence (Dolan 1984; Winn 1988; Leishman and Westoby 1994; Mojonnier 1998; Benard and Toft 2007), greater competitive ability (Gross 1984; Houssard and Escarré 1991), larger size (Verdú and Traveset 2005; Leger et al. 2009), and greater survival (Mojonnier 1998; Simons and Johnston 2000; Benard and Toft 2007) than those from smaller seeds.

We examined plant traits under selection during seedling establishment following postfire restoration in northeastern Nevada. Although seeding after wildfires is very common in the Great Basin, few seeds survive to adulthood (e.g., range of 0.5–3.4% survival across five fire sites, Kulpa 2010; also see Eiswerth and Shonkwiler 2006; James and Svejcar 2010), and thus the potential for strong selection during seedling establishment is high (Fig. 1). Alternatively, seedling establishment could be a random process, with genetic drift, rather than natural selection, responsible for changes in gene frequency during restoration. We collected seeds from the small percentage of plants that established in restored populations of a native cultivar at two fire restoration locations and compared morphological and phenological traits to those of the original restoration seed pool in common gardens, testing for changes in means and decreases in variance across generations indicative of a response to natural selection (Arnold and Wade 1984; Gomez-Raya and Burnside 1990). This method is similar to the ‘resurrection’ approach used to measure changes in gene frequency across generations for plants and dormant animals (e.g., McGraw et al. 1991; Angeler 2007; Franks et al. 2007, 2008) and is an effective way to determine if natural selection affects seedling establishment in restoration. The resurrection approach compares the gene pool before and after a selection event by growing both pools of seeds in a common garden and assumes that any significant shifts in trait distributions are the result of evolutionary change. If consistent shifts are seen in replicated selection environments, changes in gene frequency are likely due to evolution via natural selection rather than genetic drift.

We posed three related questions: (i) Have there been changes in the mean and variance of phenotypic traits during restoration? (ii) How consistent is natural selection between field sites? and (iii) What is the strength of selection on phenotypic traits during restoration? We focused our measurements on plant size, seed size, and plant phenology because these traits are commonly used to determine which seed sources will be used in restoration (Gibbs and Young 1989; Jones et al. 2004a,b). While the assumption is that larger seeds and larger plants will establish and survive better during restoration, previous research suggests that this might not be the case in highly invaded systems in the Great Basin, where natural selection can favor smaller statured plants (Leger 2008; Rowe and Leger 2011). Rapid emergence is also valued in restoration seeds (e.g., Jones et al. 2004b), and we expected that early germination would improve survival during postfire restoration, as previous experiments have documented advantages of early phenology of native plants growing in invaded systems (Leger 2008; Goergen et al. 2011).

## Methods

### Seed collection and common garden design

Two 15 ha restorations were established in Elko County, NV by the USDA Rocky Mountain Research Station, Boise, ID, in November 2006 within the perimeters of fires that burned in August 2006. One restoration was located within the East Humboldt fire (40°45′36.8″N, 115°50′22.4″W, 1585-m elevation). The invasive annual grass *Bromus tectorum* (cheatgrass) L. is present at this site, but in relatively low densities (average density of 10 plants per m^2^ in 2008, Cox et al. unpublished data). The second restoration was located within the Gopher fire (41°12′4.3″N, 115°20′59.6″W, 1710-m elevation), 60 km from the East Humboldt site. *Bromus tectorum* is widespread at this site (average density of 90 plants per m^2^ in 2008, Cox et al. unpublished data). Average precipitation at these fire sites during the establishment period of this restoration (2006–2009) was 218 mm, relative to a 30-year average of 254 mm (1980–2010, Elko WB Airport, Western Regional Climate Center 2012).

Seeded species at these two sites included the cultivar Toe Jam Creek *Elymus elymoides* (Raf.) Swezey ssp. *californicus* (J.G. Sm.) Barkworth (bottlebrush squirreltail; hereafter *E. elymoides*), seeded at densities of between 6 and 8 seeds per m^2^, among other grasses and forbs, and a subset of the exact seed used in the restoration was stored at 4°C for experimental use. We chose *E. elymoides* for this study because unlike some of the other seeded species, it established and produced seed within 2 years of planting, even in the highly *B. tectorum* invaded site. This germplasm was released for commercial use on 4 September 2003 and is intended for use in the Great Basin regions of Nevada, Oregon, Idaho, and Utah (Jones et al. 2004a). The original collection of Toe Jam Creek seed was approximately 70 km northwest from our study sites; average annual precipitation at this site is 312 mm. This particular collection of *E. elymoides* was noted to have earlier emergence and high seed mass relative to other accessions (Jones et al. 2004a).

Many *E. elymoides* plants flowered in the first summer (2007) following seeding, and all seeded plants flowered by the second growing season (2008). We collected seeds of *E. elymoides* on 25 July 2008 at both the East Humboldt and Gopher sites, from 100 individual plants per site, keeping seeds from individual maternal plants (hereafter referred to as families) separate. Seeds were stored at room temperature for 3 months. At the East Humboldt site, there were very few nonseeded native perennial grasses, but at the Gopher site, there were nonseeded *E. elymoides* plants. Drill rows and morphological differences between resident and seeded *E. elymoides* made it possible to collect seed only from seeded individuals, but it is conceivable that local plants contributed pollen to some of the seeds we collected at the Gopher site. However, due to the highly selfing nature of *E. elymoides* (Jones 1998), gene flow between resident and seeded plants was unlikely. Because we collected seeds only from mature plants growing in drill rows, seeds used in this experiment were from individuals seeded during the original restoration, thus any selection measured here likely occurred during initial establishment and juvenile phases (Fig. 1).

#### First-generation common garden

Measurements were taken on plant size and phenology in plants grown in common gardens for two generations to minimize maternal environment effects on phenotypes caused by differences in growing conditions between the original restoration seed and field-collected seed. For the first common garden, four seeds from each of the 100 maternal family lines from each field site and 400 seeds from the original restoration seed were haphazardly selected and individually weighed without awns. Each F1 seed was glued to a separate toothpick using Elmer's Washable School Glue (Elmer's Products Inc, Columbus, OH) before planting, to aid in tracking performance of individual seeds. A total of 1200 seeds were planted on 15 October 2008 in an outdoor common garden at the University of Nevada Reno (hereafter, F1 garden) with individual seeds randomly assigned to locations within a fully randomized design. Average growing season precipitation at the common garden site is 183 mm (2000–2011, UNR Valley Road Farm, Western Regional Climate Center 2012). Seeds were planted at 30 cm spacing on alternating sides of a row-line, and weeds were removed by hand throughout the growing season. One seed from the original restoration source proved to be a different *Elymus* species (likely a seed contaminant) and thus 1199 seeds were included in the final analysis.

Seeds were monitored for emergence immediately after the first fall rains (the typical time for grass seed emergence) and as winter weather permitted throughout the winter, for a total of nine seedling censuses: 25 and 29 November 2008; 4, 11, and 30 December 2008; 10 January 2009; 4 and 20 February 2009; and 12 March 2009. Morphological and phenological traits were measured in the spring and summer of 2009 and 2010. Plant height (cm), leaf number, and spikelet number were measured on 16 June 2009, and plant height and basal area (length by width) were measured on 2-year old plants in July 2010. Plant heights and leaf numbers were also collected in April and May, but as these results were identical to collections in June, results are not presented. Mature seeds were collected weekly between 16 June 2009 and 18 August 2009, and total reproductive biomass was determined for each plant by weighing the total seeds produced, and phenology of seed production was characterized by determining the percentage of total seed produced in June, July, and August 2009. Due to the large volume of seeds produced in 2010, seeds were not collected in the second year of growth. Flowering phenology of all plants was tracked in the second year of growth, with daily monitoring between 12 April 2010, when the first plants flowered, and 22 May 2010, when the last plant flowered. Above-ground vegetative biomass was harvested on 18 August 2009. Plants were dried at 40°C for 5 days and the mass of total vegetative biomass was recorded for each plant. In 2010, plant biomass was harvested for a subset of 10 plants on 16 July 2010, to verify that basal area measurements (taken for all plants) were correlated with biomass.

#### Second-generation common garden

A randomly selected subset of 50 families originating from both field sites and the original restoration source (150 total families) were planted in a second common garden in 2009 (hereafter, F2 garden). Eight seeds per family were individually weighed, and four seeds per family were glued on toothpicks and planted adjacent to the F1 garden on November 3, 2009. These seeds were produced in a common garden where gene flow between the original seeds and those collected from the field sites would have been possible, but due to the highly selfing nature of these plants, this was unlikely, though any gene flow would be expected to increase similarity among original and field-collected seeds in the F2 generation. Seedling emergence was later and more synchronous in the 2009–2010 growing season than in 2008–2009, and we monitored daily after germinating rains from 11 February 2010 through 1 March 2010. Flowering began on 14 May 2010 and was tracked daily through 3 July 2010, after which no other plants flowered. Seeds were collected every 2–3 days from 29 June through 2 August 2010. Total seed production was measured by summing the total seed production from all collection dates, and phenology of seed production was characterized by summing seeds produced from 29 June to 7 July (early), 9 July to 19 July (mid), and 22 July to 2 August 2010 (late). Plant height and spikelet number were measured on 25 June 2010. Biomass measurements and leaf numbers showed nearly identical patterns in the F1 common garden, and thus, only biomass measures were taken in the F2 garden. Above-ground vegetation was harvested on 9 August 2010; plants were dried and weighed as above. Eight seeds per plant were randomly selected and weighed individually to generate F3 seed sizes.

## Data analysis

Unless specified, all analyses were conducted using JMP version 9 statistical software (SAS Institute Inc., Cary. NC); values presented in text and figures are untransformed means and standard errors, unless otherwise noted. anova was used to test the main effects of seed source (either original restoration seeds or field-collected seed), and site (nested within source) for F1 garden response variables. In addition, differences in timing of first-year seed production among sources and sites were analyzed with manova, using the Wilk's *λ* method of determining significance, with the percentage of seeds produced early, mid, and late season in 2009 as response variables; significant differences among sources and sites were subsequently analyzed with anova. Because family structure was only present in field-collected seeds in the F1 garden, a second model was run to test for family-level differences in all measured traits for plants from the two field sites only. This mixed model included collection site and the random effect of family (nested within site). Plant height, number of leaves, number of spikelets, and total weight of seed produced were transformed with the Box Cox transformation to meet the assumptions of homogeneity of variances and normal distribution of residuals; F1 and F2 seed size, emergence day, and number of leaves were log-transformed; other variables did not require transformation to meet assumptions of anova.

Logistic regression was used to compare emergence and survival between sources and collection sites (nested within source), with status at the end of the growing season (alive, emerged by dead, or nonemergent) as the response variable. Logistic regression was used to test the effect of emergence timing on first-year survival, with survival as a response variable and source, site (nested within source) and emergence date as model factors. The effect of seed size on emergence and first-year survival and emergence was analyzed using logistic regression, with the response variable of final plant status (alive, dead, nonemergent) and the effects of source and site (as above), as well as seed size, and their interactions. Linear regression was performed between seed size and timing of seedling emergence.

In the F2 garden, analyses were similar except that the random family factor was included for all three seed sources in all anovas. In addition, changes in seed size and plant biomass between the two gardens were analyzed with a model that included seed source, site (nested within source), generation, and the site × generation interactions. Flowering time was transformed with the Box Cox transformation, and F3 seed size, the number of spikelets, and total seed production were log-transformed for analysis. Means comparisons among seed sources were conducted using Tukey's adjusted least square means for multiple comparisons. Differences in variance among sites, for all traits in both common gardens, were compared using the nonparametric ANOM (analysis of means method, Nelson et al. 2005) for variance with Levene's method, which determined if site variance deviated from the overall root mean square error.

The selection differential, *S*, was calculated by comparing the mean values of each trait in the original seed source with the mean of the values from the field-collected plants (combined when mean values were not significantly different between field sites), and the standardized linear selection differential *i* (the intensity of selection) was calculated by standardizing the selection differential for each phenotypic trait (Falconer and Mackay 1996). The standardized differential is calculated so comparisons can be made among traits within this experiment and with measures of *i* from other studies (e.g., Kingsolver et al. 2001; Geber and Griffen 2003). *S* and *i* were calculated as follows:

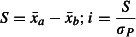
 where 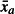 is the mean of a given trait after selection (the mean of the field-collected population), 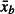 is the mean of that trait before selection (the mean of the original source population), and *σ*_*P*_ is the standard deviation of the phenotype (Falconer and Mackay 1996). Pearson's partial correlation coefficients were calculated among traits in the F1 and F2 common gardens to determine the degree of associated among pairs of traits using PROC CORR in SAS version 9.3.

## Results

### Seed size

**Figure 2:**
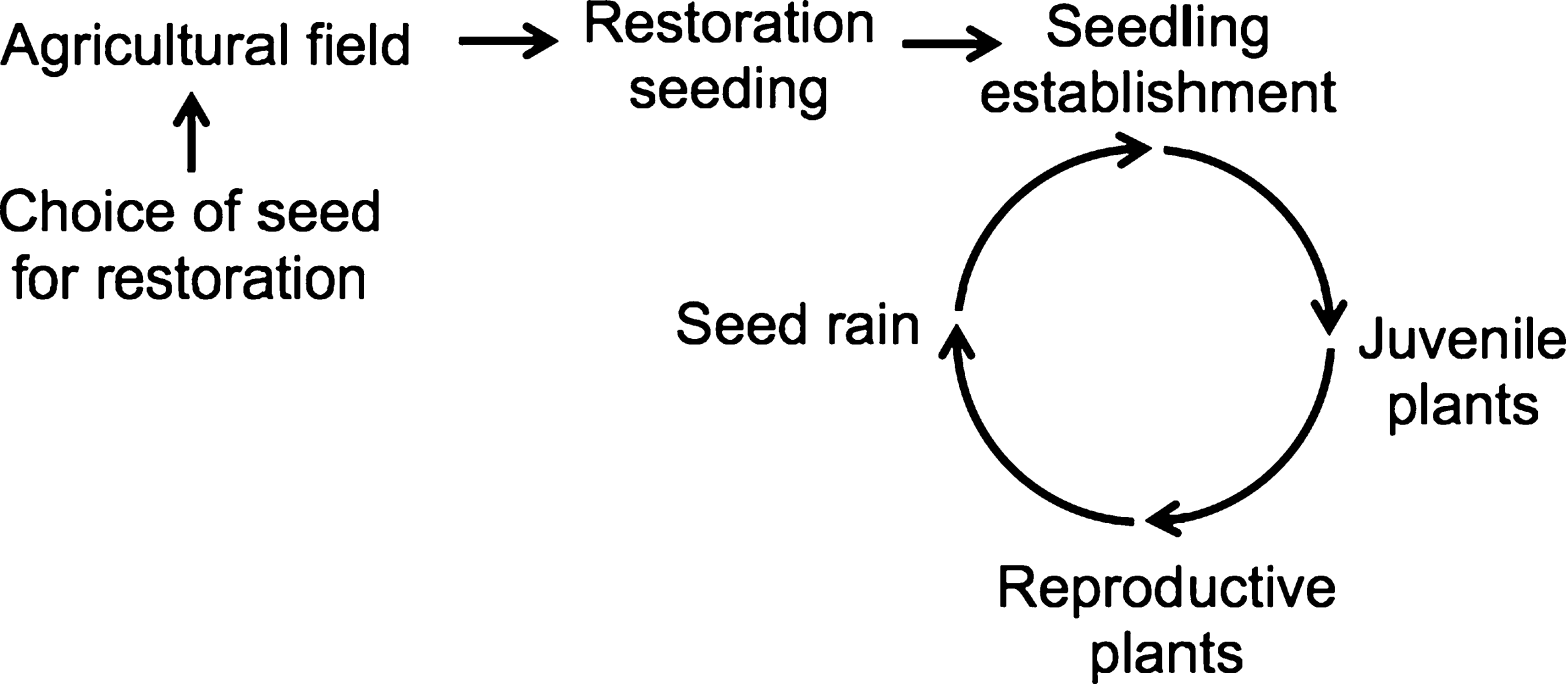
Distribution of seed weights among sources of *Elymus elymoides* collected from two field restorations (East Humboldt and Gopher) and the original restoration source. Columns represent seed weights from the original restoration seed and field-collected seeds from field sites (F1), seeds collected from the first-year common garden (F2), and seeds collected from a second common garden grown from F2 seeds (F3). Means are indicated with a bold line, and values are means (x), standard deviation (*σ*), and selection differentials (*i*). Letters indicate significant differences among sources based on Tukey's HSD tests, conducted separately for F1, F2, and F3 seeds.

Seed size differed significantly among sources, for F1, F2, and F3 generations (Table 1A). Seeds from the original restoration source were significantly larger than seeds collected from the two rehabilitation sites in all generations (Fig. 2). Only F1 seed size differed between the two field sites (Table 1A): Seed from the Gopher site was initially smaller than East Humboldt, but seed sizes between the two sites converged to nearly identical means in the F2 and F3 generations (Fig. 2). Seed size changed over time for some sites but not others (generation × site interaction, *F* = 39.6_(2, 4502)_, *P* < 0.0001). Plants from the East Humboldt site showed no change in seed size over time, plants from the Gopher site showed a significant increase of 23.1% between F1 and F2 and no change between F2 and F3, whereas seeds from the restoration source showed a small but significant decline of 4.2% and 7.1% between F1 and F2 and between F2 and F3, respectively. In all three generations, seed sizes differed significantly among families (Table 1A).

**Table 1:** anova and manova (timing of seed set) results of measures of plant performance in *E. elymoides* from a common garden experiment in Reno, NV

		Source		Collection Site (Source)		Family (Collection Site)	
				
		*F*	*P*	*F*	*P*	*F*	*P*
A. Seed size	Fl seed size	1435.3_(1, 1196)_	**< 0.0001**	158.0_(1, 1196)_	**< 0.0001**	8.1_(198, 600)_	**< 0.0001**
	F2 seed size	238.6_(1, 140)_	**< 0.0001**	0.01_(1, 140)_	0.9938	7.4_(139, 1055)_	**< 0.0001**
	F3 seed size	337.7_(1, 146.9)_	**< 0.0001**	0.09_(1, 147)_	0.859	7.1_(146, 1046)_	**< 0.0001**
B. Size and reproduction	F1 height	71.14_(1, 444)_	**< 0.0001**	0.85_(1, 444)_	0.3375	0.27_(155, 124)_	0.6037
	F1 number of leaves	62.20_(1, 444)_	**< 0.0001**	0.31_(1, 444)_	0.5772	0.98_(155, 124)_	0.5574
	F1 number of spikelets	0.10_(1, 444)_	0.7502	0.47_(1, 444)_	0.4923	0.86_(155, 124)_	0.8143
F1 biomass	304.11_(1, 444)_	**< 0.0001**	1.65_(1, 444)_	0.1991	1.10_(155, 124)_	0.2888
F1 basal area (2010)	28.67_(1, 430)_	**< 0.0001**	0.03_(1, 430)_	0.086	0.97_(151, 188)_	0.5106
F2 height	19.7_(1, 139)_	**< 0.0001**	0.09_(1, 253.5)_	0.7585	0.71_(137, 189)_	0.9834
F2 number of spikelets	0.16_(1, 169.7)_	0.6904	0.01_(1, 242.2)_	0.9478	1.1_(137, 188)_	0.8893
F2 biomass	24.1_(1, 172.5)_	**< 0.0001**	0.12_(1, 244.96)_	0.7293	0.84_(138, 190)_	0.8654
F1 height	71.14_(1, 444)_	**< 0.0001**	0.85_(1, 444)_	0.3375	0.27_(155, 124)_	0.6037
C. Phenology	F1 emergence time	1.7_(1, 787)_	0.1925	0.01_(1, 787)_	0.9781	1.13_(176, 365)_	0.1722
	F1 flowering time (2010)	84.8_(1, 431)_	**< 0.0001**	21.17_(1, 431)_	**< 0.0001**	2.06_(155, 121)_	**< 0.0001**
	F1 timing of seed set	24.5_(3, 372)_	**< 0.0001**	3.88_(3, 372)_	**0.0095**	–	–
F2 emergence time	0.3_(1, 151.2)_	0.5891	0.01_(1, 165.5)_	0.9769	1.01_(146, 380)_	0.4599
F2 flowering time	0.3_(1, 54.5)_	**< 0.0001**	9.0_(1,53.1)_	**0.0031**	1.3_(133, 150)_	0.0540
F2 timing of seed set	15.2_(3,135)_	**< 0.0001**	1.3_(3, 135)_	0.2727	1.0_(399, 405)_	0.3304

Measurements from the F1 garden were taken in 2009 and 2010 (responses are for 2009 unless otherwise specified) and in 2010 for the F2 garden. In the F1 garden, family was analyzed for field-collected seeds only, but family was included in all F2 and F3 measurements. Numbers in parentheses are numerator degrees of freedom, denominator degrees of freedom. Bold values highlight significant (*P* < 0.05) results.

### Plant size and reproduction

**Figure 3:**
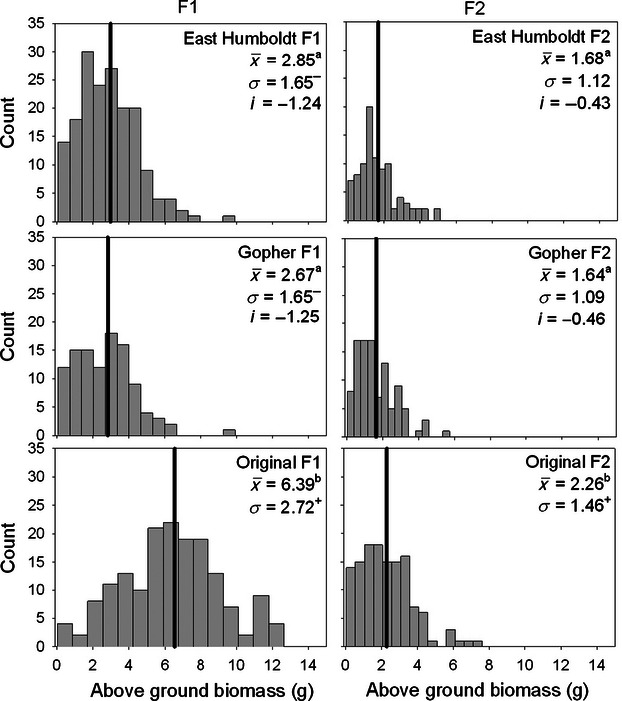
Distribution of end of season above-ground biomass of first-year seedlings among three different sources of *Elymus elymoides* in the F1 (first column) and F2 (second column) common garden experiments. Means are indicated with a bold line, and values are means (x), standard deviation (*σ*), and selection differentials (*i*). Letters indicate significant differences among sources based on Tukey's HSD tests, conducted separately for F1 and F2 biomass.

In the both the F1 and F2 gardens, plants from the original restoration source were consistently larger and made more seeds than plants from either of the field sites, whether the response variable was above-ground biomass, plant height, leaf number, basal area, or total seed production (Table 1B, Fig. 3). Plants from the two field sites showed consistent trait means, with no significant differences between plants collected from Gopher or East Humboldt for any size or reproduction variable (Table 2). In general, the 2009–2010 growing season was less favorable for plants than the 2008–2009 growing season (153 mm of precipitation received in 2009–2010 growing season vs 533 mm in 2008–2009). Average biomass for 1-year old plants in the F2 garden was 2.10 ± 0.07 g, relative to 4.27 ± 0.13 g for 1-year old plants in the F1 garden. Although the plants from all locations were smaller in the F2 garden, plants from the restoration source declined more in response to less favorable growing conditions (significant source × generation interaction, *F* = 121.2_(1, 710)_, *P* < 0.0001), with a 43.6% and 42.6% decline in vegetative biomass observed in plants from East Humboldt and Gopher, respectively, and a 65.0% decline in size of plants from the restoration source (Fig. 3). No size or reproductive output traits varied significantly among families (Table 1B).

**Table 2:** Traits of *E. elymoides* measured in F1 and F2 common gardens that are not shown in figures

		Source			
			
Generation	Trait	Original seed	East Humboldt	Gopher	Selection differential (*i*)
F1						Height (cm)	26.1^a^ (6.77)	21.1^b^ (6.46)	20.3^b^ (6.53)	−0.74
Number of leaves	98.9^a^ (44.5^+^)	65.4^b^ (35.9^−^)	68.8^b^ (39.0)	−0.76
Number of spikelets	3.5^a^ (4.07)	3.5^a^ (4.24)	2.8^a^ (3.34^−^)	–
Total seed produced (g)	1.2^a^ (0.98^+^)	0.6^b^ (0.74)	0.4^b^ (0.47^−^)	−0.82
Basal area (cm^2^) (2010)	72.5^a^ (24.95)	59.7^b^ (22.31)	59.2^b^ (26.01)	−0.53
Emergence timing	17.3^a^ (23.94)	19.7^a^ (26.55)	22.9^a^ (31.4^+^)	–
F2					Height (cm)	23.5^a^ (7.53)	20.8^b^ (5.83)	20.0^b^ (6.0)	−0.46
Number of spikelets	5.6^a^ (4.45)	5.8^a^ (4.14)	5.6^a^ (4.61)	–
Total seed produced (g)	2.2^a^ (1.37^+^)	1.5^b^ (0.91)	1.3^b^ (0.77^−^)	−0.75
Emergence timing	2.8 (2.9)	2.7 (3.0)	2.7 (3.4)	–
Timing of flowering	19.7^a^ (7.2)	11.6^b^ (7.5)	14.6^c^ (8.4)	−0.97, −0.62

Values are means (standard deviations); lower case letters indicate significant differences among means based on Tukey's HSD, and +/− indicate when variances are significantly higher or lower than expected, based on Levene's tests. Selection differentials were calculated when there was a difference between the original and field-collected seeds. When field sites did not differ significantly from one another, differentials were based on pooled means/standard deviations and were calculated separately for each field site when they differed.

### Phenological traits

**Figure 4:**
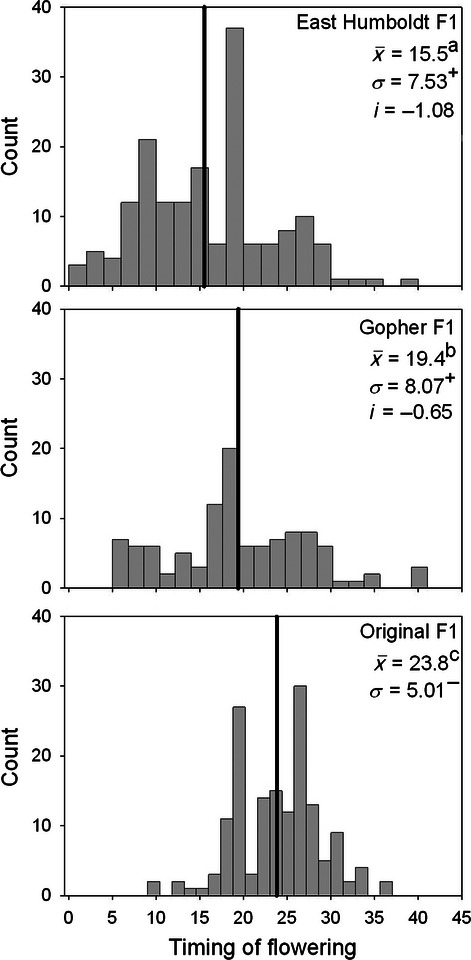
Distribution of flowering time in the second year of growth for three sources of *Elymus elymoides* in the F1 common garden. Means for each of the collection are indicated with a bold line, and values are means (x), standard deviation (*σ*), and selection differentials (*i*). Letters indicate significant differences among sources based on Tukey's HSD tests.

**Figure 5:**
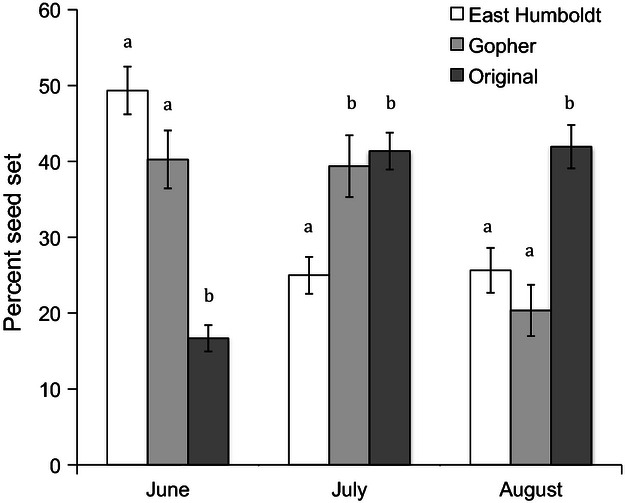
Average seed set by month for first-year plants from three sources of *Elymus elymoides* in the F1 common garden. Means and standard errors are shown, and letters indicate significant differences among sources based on Tukey's HSD, calculated separately within months.

Emergence time did not differ by sources, sites, or families, in either the F1 or F2 garden (Table 1C). In contrast, sources and collection sites differed in flowering time in both the F1 and F2 gardens (Table 1C). In the F1 garden, 2-year old plants from East Humboldt flowered earliest, followed by plants from Gopher, and the original restoration seed flowered latest (Table 2, Fig. 4). Differences in flowering phenology between sources and populations remained significant in the F2 garden (Tables 1C and 2). Similarly, there was a shift in the timing of seed production of 1-year old plants among the original and field-collected seeds in both gardens (Table 1C). Field-collected seeds in the F1 garden had the highest seed production in June and July, while seed production in the original source seeds was primarily in July and August, with minimal seed production in June (Fig. 5). There were differences between the two field collection sites, with East Humboldt plants setting a larger proportion of seeds in June than plants from Gopher, a site where seed production was similar between June and July (Fig. 5). In the F2 garden, plants also showed significant differences in seed set between sources, but not sites (Table 1C). As in the F1 garden, seed set was earlier for the field-collected seeds than for the original seeds (field-collected seeds early seed set 74.7% ± 2.3; original source early seed set 56.7% ± 2.9).

### Emergence and survival

Overall emergence in the F1 garden was 67.9%, with 815 of 1199 seeds emerging. Of emergent seeds, 487 plants (59.8%) survived through the end of the first growing season, and 434 (36.2%) survived through the end of the second growing season. Emergence and first-year survival differed by source (*χ*² = 19.8_2_, *P* < 0.0001) and among collection sites (*χ*² = 45.0_2_, *P* < 0.0001), differences that persisted through the second growing season (*P* < 0.0001). The greatest emergence and establishment was observed in the seeds collected from the East Humboldt site (80.8% emergence, 47.3% establishment), intermediate values were observed in the original source (63.4% emergence, 43.4% establishment), while seeds from the Gopher site displayed the lowest emergence and survival (59.8% emergence, 31.3% establishment). Seedling death between the first and second growing seasons was similar among the collection sites, with 5.0%, 4.0%, and 4.3% of first-year seedlings dying between 2009 and 2010 for East Humboldt, Gopher, and the restoration source, respectively.

In the F2 garden, overall emergence was 89.2%, with 535 of 600 seeds emerging. Of emergent seeds, 328 (60.7%) survived through the end of the first growing season. As in the F1 garden, emergence and first-year establishment differed among sources (*χ*_2_² = 16.9_2_, *P* = 0.0002) but not sites (*χ*² = 1.8_2_, *P* = 0.4124). Seeds from the original restoration source were more likely to emerge and survive (92.5% emergence, 66.5% establishment) than seeds from East Humboldt (86.0% emergence, 45.5% establishment) or Gopher (88.9% emergence, 51.8% establishment).

Early emerging seeds had greater first-year survival in the F1 garden (*χ*² = 11.4_1,_ *P* = 0.0007), an effect that persisted through the second year of growth (*χ*² = 11.1_1,_ *P* = 0.0008). Early emerging seeds had greater survival in the F2 common garden as well (*χ*² = 10.7_1_, *P* = 0.0011). Seeds that emerged, died, or survived differed significantly in seed weight in the F1 garden (*F* = 49.2_(2, 1190)_, *P* < 0.0001) and F2 garden (*F* = 3.3_(2, 591)_, *P* = 0.0384), and there was an interaction between seed size and source (*F* = 16.5_(2, 1190)_, *P* < 0.0001) in the F1 garden. In the original restoration source, seeds that emerged and survived (4.02 ± 0.05 mg) were significantly larger than seeds that emerged and died (3.7 ± 0.09 mg) and nonemerging seeds (3.74 ± 0.06 mg), but for the field source, there was not a difference in seed size between emergent plants that were alive or dead (alive: 2.3 ± 0.03 mg, dead: 2.2 ± 0.03 mg), though nonemerging seeds (1.6 ± 0.04 mg) were significantly smaller than emergent ones. Larger seeds emerged significantly faster than smaller seeds in the F1 (*F* = 9.04_(1, 784)_, *P* = 0.0027) and F2 (*F* = 6.6_(1, 524)_, *P* = 0.0107) gardens, but the explanatory power of seed size to predict emergence timing was low (F1, *R*^2^ = 0.0134; F2, *R*^2^ = 0.0036).

### Variation, correlation among traits, and selection differentials

Levene's tests indicated that variances were significantly higher in the original source (all *P* < 0.05) than in field-collected seed for F1, F2, and F3 seed size (Fig. 2), F1 and F2 biomass (Fig. 3), and in the F1 garden for the number of leaves and total seed production (both years, Table 2). F1 emergence and flowering phenology were two cases where field-collected seeds showed greater variation than the original source (Table 2). The Gopher and East Humboldt sites also differed from each other in the variance of some traits (Table 2), with one site or the other showing decreased variance relative to the data set as a whole.

Partial correlations indicated significant relationships among traits in the F1 and F2 gardens. Seed size was positively correlated with flowering time in both gardens (plants grown from larger seeds flowered later), positively correlated with biomass, negatively correlated with leaf and spikelet number in the F1 garden, and positively correlated with total seed production in the F2 garden (Appendixes A and B). Positive correlations between measures of seed production (e.g., number of spikelets and total seed production) and measures of plant size (e.g., height and biomass) were observed in both gardens. Flowering time and spikelet number were negatively correlated in the F2 garden, with later flowering plants making fewer spikelets (Appendix A). Emergence timing was not correlated with any trait in either year (Appendixes A and B).

All standardized selection differentials were negative, with an average of −1.0 ± 0.1 across all traits. Selection differentials were highest for seed size (between −1.42 and −1.66, Fig. 3) and plant size (between −0.54 and −1.34, Fig. 4), followed by flowering phenology in the F1 (Fig. 4) and F2 gardens (Table 2). Selection differentials were between −0.46 and −0.82 for other size and seed production measures (Table 2).

## Discussion

Postfire seeding in the Great Basin occurs across vast spatial scales, and the use of local propagules is typically not a component of these restorations due to the scarcity of local seeds and the abundance and lower cost of cultivars (Jones and Johnson 1998; Richards et al. 1998; Jones and Larson 2005). Early emergence, large plant and seed size, and high seed production are often selected for in cultivar development (e.g., Gibbs and Young 1989; Jones et al. 2004a,b), presumably because these traits increase seed production in agricultural settings, as well as for the perceived benefits of these traits in natural systems. In our study, however, we found that the traits most associated with success were different from those selected during cultivation.

We found evidence for strong directional selection during two ecological restoration projects, illustrated by changes in the distribution of phenotypes of plants that survived in restored field sites relative to that of the original seed used for restoration. Survival was greater for plants that possessed a correlated suite of traits, namely smaller seed and plant size, and early flowering phenology. Despite the relatively local origin of the native cultivar seeds used in this experiment, means and variances of nearly every trait measured changed significantly after restoration, and these differences persisted through multiple generations, indicating that evolutionary shifts rather than maternal environment effects were responsible for changes in phenotype. We observed convergence of morphological traits between our two restoration sites, indicating that observed changes were most likely an evolutionary response to selection rather than a consequence of genetic drift. Similar shifts in plant size were observed when a subset of F2 seeds were grown in a greenhouse environment (Ferguson 2012), indicating that the observed size differences are robust to different growing environments.

Selection differentials were large relative to those measured in wild and experimental populations in other systems (Kingsolver et al. 2001; Geber and Griffen 2003). Both of these published reviews analyzed the strength of natural selection in the wild from hundreds of studies of plants and animals (Kingsolver et al. 2001), or plants in the wild and in experimental studies (Geber and Griffen 2003). Values of *i* reported by Kingsolver et al. (2001) varied between −1.0 and 1.5, with most values between −0.5 and 0.5 (only two studies with values less than −0.5), and the absolute value of *i* was 0.27 ± 0.16 for plant studies (Geber and Griffen 2003). The magnitude of *i* values observed in our study, (−1.0 ± 0.1) highlights an extreme lack of fit between the frequency of traits in the restoration source pool and optimal phenotypes in the restoration site. Whether this magnitude of mismatch we observed is unusual in plant restorations is unknown, but comparisons with other, similar studies conducted in different habitats would be instructive.

What might the mechanisms of increased survival be for smaller statured, smaller seeded, and earlier flowering plants? A variety of biotic (e.g., predation, competition, disease) and abiotic (e.g., climate, resource availability) environmental conditions in the restoration field sites could constrain the establishment of planted seeds. Limited water, and thus limited access to soil resources, is almost certainly a strong selective agent in desert systems, and other research in these systems has demonstrated that increased seedling allocations to roots (Rowe and Leger 2011), early adult phenology (Leger 2008; Goergen et al. 2011), and small plant size (Rowe and Leger 2011) can be adaptive in highly invaded arid systems. Precipitation at the restoration sites was below the average precipitation at the source population (218 mm during the restoration period vs 312 mm average at the collection site), and the presence of *B. tectorum* at the restoration sites is likely to have limited plant-available water even further. Thus, water limitation may have been a strong selective force during this restoration. We cannot yet determine which traits are increasing survival in these restorations. Strong correlations among traits in our study could indicate the possibility for pleiotropic effects, with selection on one of our measured traits (or an unmeasured trait, such as resource use efficiency, allocation to roots, or resistance to natural enemies) driving changes in multiple traits, or constraints of genetic architecture could result in correlated changes among gene regions (Falconer and Mackay1996; Blows and Hoffmann 2005; Carroll 2007). However, research in other systems links seed size, plant size, and phenology with plant fitness; thus, we discuss hypotheses about how these traits may be adaptive in our system.

Of these correlated traits, seed size showed the strongest selection differentials and differentiation among families. A genetic basis for seed size has been observed in many plant species (e.g., Voigt et al. 1966; Drabo et al. 1984; Malhotra et al. 1997; Upadhyaya et al. 2006), and the evolution of seed size has been studied extensively, often in the context of trade-offs in maternal provisioning (Stebbins 1971; Smith and Fretwell 1974; Venable 1992). Maternal environmental effects can also be nongenetic contributors to seed size (Roach and Wulff 1987). In our experiment, differences in seed size remained constant over multiple generations of growth in a common environment, and the remarkably consistent distribution of seed sizes in field-collected plants after the F1 generation (where increased competition from *B. tectorum* at the Gopher site may have led to a maternal environment cause for the initially lower seed sizes) indicate a strong genetic contribution to seed size in these plants.

There are two ways that seed size may have affected field performance: first, through direct effects on seedling size and germination timing, and second, by indirect effects of seed predators. As discussed above, larger seed size has been shown to improve plant performance in many systems (Verdú and Traveset 2005), but other studies have shown that large seed size can have positive or negative effects on survival, depending on environmental conditions (e.g., Hendrix and Trapp 1992; Paz et al. 1999; Parciak 2002). In our common garden, seed size did influence emergence timing and survival, with larger seeds emerging earlier and surviving better from all collection sites; however, the response to selection in the restoration sites clearly shows higher survival of smaller seeded plants (Fig. 2). This may be an evidence of a genotype by environment interaction, such that larger seeds perform better in common garden conditions, benefiting from either the reduction in competition, herbivory, or disease relative to the restoration sites. Smaller seeds may be better able to avoid predation, which may be particularly relevant in postfire restorations in the Great Basin where diversity and abundance of rodents can increase after disturbance (Longland 1996). Predation can be higher on larger seeds, either because they are more apparent to herbivores or because they are selected by herbivores to a greater degree (Vander Wall 1994; Hoffmann et al. 1995; Celis-Diez et al. 2004), and selective predation can affect the recruitment of individuals to populations (e.g., Bricker et al. 2010). Increased seed production may also incur physiological costs, as plants that invest more in reproduction have shorter life spans than plants with more conservative reproductive strategies (Bender et al. 2000; Obeso 2002). Because we collected seeds from young reproductive individuals, this was unlikely to be a strong selective force during this experiment (Fig. 1), but could negatively affect long-term survivorship of plants with greater reproductive allocation.

Not only were seed sizes smaller in plants that survived in the field, but plant sizes were also smaller than the mean of the original source, again with these differences persisting through multiple generations (Fig. 3). While size and fitness are often assumed to be directly related for plants, some evolutionary strategies favor small plants over large ones, evidenced by a right-skewed distribution of species sizes in plant communities, comparable with that observed for animals (Aarssen et al. 2006). Increased allocation to reproductive, rather than structural, tissues has been proposed as one explanation for the abundance of small plant species, but there may be physiological reasons why small plant size is adaptive in arid environments. Reduced leaf area, and thus reduced transpiration, is a possible a mechanism for increased performance of smaller individuals in dry environments. In agriculturally important grasses like wheat, small plants are less affected by drought stress than larger plants (Blum and Sullivan 1997), a phenomenon also observed in dwarf sunflowers (Angadi and Entz 2002). Similar results have been observed for wild plants under drought conditions: Smaller plants can have increased performance relative to larger plants, increasing in size and overtaking larger plants (Casper 1996), and smaller seedlings can survive short-term droughts better than larger seedlings (Hendrix et al. 1991). The original seed source showed a greater reduction in above-ground size in the second, less favorable year, which may indicate a differential ability to tolerate environmental stress. An arid climate and strong resource competition from invasive annual grass may have been responsible for the increased survival of smaller statured plants in our restoration sites.

There were also differences in phenology between plants from the field and the original restoration seed: Mean shifts were observed in flowering time, and phenological traits were the only cases where variance was higher in field-collected seeds than the original source seeds. Maternal environment in the field may have increased variation in these particular traits, as an example of a nongenetic influence on trait expression passed on from parent to offspring (Galloway et al. 2009). Plants from the two field sites in the common garden set the majority of their seed earlier in the season (June and July), while plants from the original restoration material set the majority of their seed late in the growing season (August). Earlier flowering phenology is often seen in dry environments with little summer rain (Rathcke and Lacey 1985; Rice and Mack 1991; Hall and Willis 2006; Petrů et al. 2006) and is likely adaptive because it ensures greater access to water resources before plants go dormant for the summer (Volaire and Norton 2006). Strong selection for early flowering in response to climate drying has been observed in other systems (e.g., Franks et al. 2007) and was found to be adaptive for many plant species, especially those in temperate climates (Munguía-Rosas et al. 2011). Emergence phenology can also affect plant fitness (Rathcke and Lacey 1985; Verdú and Traveset 2005). In our experiment, plants that emerged earlier had greater survivorship than later emerging plants, consistent with other studies (Cook 1980; Benard and Toft 2007). We would have predicted a trait shift toward early emergence in the field-collected seeds, but this did not occur. There was no evidence for genetic variation in emergence time in the original restoration source (Table 1) nor was emergence time correlated with any other trait (Appendix A, B), thus lack of variation may have prevented an evolutionary response to selection.

Although ecological restorations and studies of natural selection in the field are common, studies that combine the study of natural selection and restoration are not. Substantial benefits to restoration could result from understanding how genetic factors affect establishment, and the opportunity to study natural selection at large scales is an untapped opportunity for understanding evolution in complex environments. Our experiment illustrated that natural selection can play a strong role in restoration projects, and that small, early flowering plants were the most successful at establishing at two restoration sites in this arid system. The need for off-site, agricultural increase of seeds for restoration creates a situation in which artificial selection may run counter to natural selection. In some cases, agricultural field production selects for traits that are directly opposed from those that increase fitness in natural environments. To improve restoration success, feedback must be established between plant performance in the wild and artificial selection in the plant development phase of restoration. The methodology employed here, wherein seeds are stored from a parental generation and the ‘resurrected’ for comparison with subsequent generations, is increasing in use in evolutionary studies (e.g., Jensen et al. 2012), and will be the foundation of a large seed collection effort designed to quantify plant responses to climate change (Franks et al. 2008). This method could be employed easily in restoration, increasing our understanding of how traits affect fitness in complex landscapes.

## Data archiving statement

Data for this study available at Dryad - doi:10.5061/dryad.10453

## Supporting Information

**Appendix A.** Pearson's partial correlation coefficients among traits measured in the F1 common garden. **Appendix B.** Partial correlation coefficients among traits measured in the F2 common garden.
