# Supplementary material for: Strong natural selection during plant restoration favors an unexpected suite of plant traits
Source: Evol Appl. 2013 Jan 3;6(3):510–23. doi: 10.1111/eva.12038 (PMC3673478; doi:10.1111/eva.12038)
Supplement: Supplementary file 1 — Appendix A. Pearson's partial correlation coefficients among traits measured in the F1 common garden. Appendix B. Partial correlation coefficients among traits measured in the F2 common garden. [file eva0006-0510-sd1.docx]

**SUPPORTING INFORMATION**

**Supplementary Text S1: Validation of coarse-grained approach by using all atom Molecular Dynamics**

**Replica Exchange Molecular Dynamics.** The protein structure is seeded into the replica exchange molecular dynamics (REMD) analysis (Sugita and Okamoto 1999) simulation with the temperature of the replicas ranging from 270K to 450K. Each structure is run using the amber force field with a GBSA implicit solvation model (Tsui and Case 2000) and a SA penalty term of 5 cal/mol A°. Swaps between structures are attempted every 1ps. For these runs, we attempt to swap replicas five times in each swap step. The swap ratio is approximately 0.52, with the goal being 0.50. Each structure is run for 5ns per replica to achieve convergence by checking the Pearson correlation coefficient between the global fluctuation profiles (i.e. the slowest modes) of two covariance matrices obtained in different window sizes (i.e. agreement between the lowest normal modes obtained from 3-4 ns and 4-5 ns MD trajectories) (Glembo et al. 2012).

**Essential dynamic analysis on all-atom REMD trajectories.** Upon completion of the REMD simulations, the lowest temperature replica is analyzed with PCA (Hayward and de Groot 2008)**.** An alpha-carbon covariance matrix is calculated from a 2ns window of the trajectory. In order to calculate the covariance matrix, the rotational and translational motion of the protein is first removed by superimposing the conformational snapshots. A matrix, X_n_, is then generated for the sampling window. The inverse of the mass weighted covariance matrix is related to the second order derivative of the elastic energy potential (i.e. harmonic Hamiltonian), which is called Hessian matrix (Atilgan et al. 2001). To incorporate the MD covariance information into PRS, the Hessian matrix calculated from the connectivity is replaced by the inverse of the covariance matrix calculated from a PCA of the MD trajectory. After the insertion of the MD covariance matrix, the standard PRS method is completed with sequential residue perturbations. The *dfi* values are then extracted as explained in the method section of the main manuscript.

Atilgan, A. R., S. R. Durell, R. L. Jernigan, M. C. Demirel, O. Keskin, and I. Bahar. 2001. Anisotropy of fluctuation dynamics of proteins with an elastic network model. Biophysical Journal 80:505-515.

Glembo, T. J., M. F. Thorpe, D. W. Farrell, Z. N. Gerek, and S. B. Ozkan. 2012. Collective Dynamics Differentiates Functional Divergence in Protein Evolution. PLoS Computational Biology 8:e1002428.

Hayward, S. and B. L. de Groot. 2008. Normal modes and essential dynamics. Methods in Molecular Biology **443**:89-106.

Sugita, Y. and Y. Okamoto. 1999. Replica-exchange molecular dynamics method for protein folding. Chemical Physics Letter*s* **314**:141-151.

Tsui, V. and D. A. Case. 2000. Theory and applications of the generalized Born solvation model in macromolecular simulations. Biopolymers **56**:275-291.


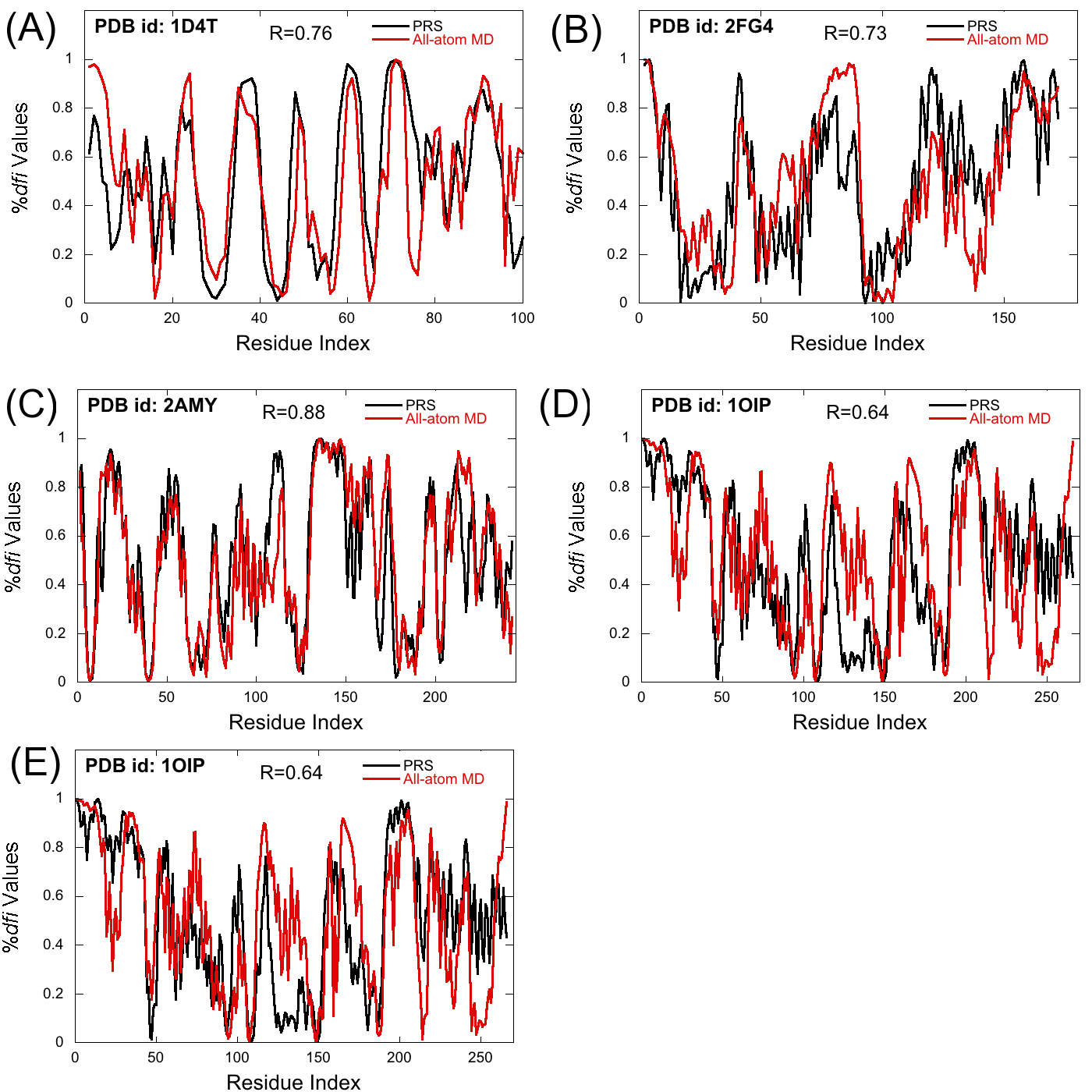


**Figure S1.** Plots of %*dfi* values obtained from our coarse-grained PRS approach are in black, *dfi* values predicted with all-atom REMD trajectories in red. The Pearson correlation coefficients between these two estimates are given in plots. These two estimates show high correlations.


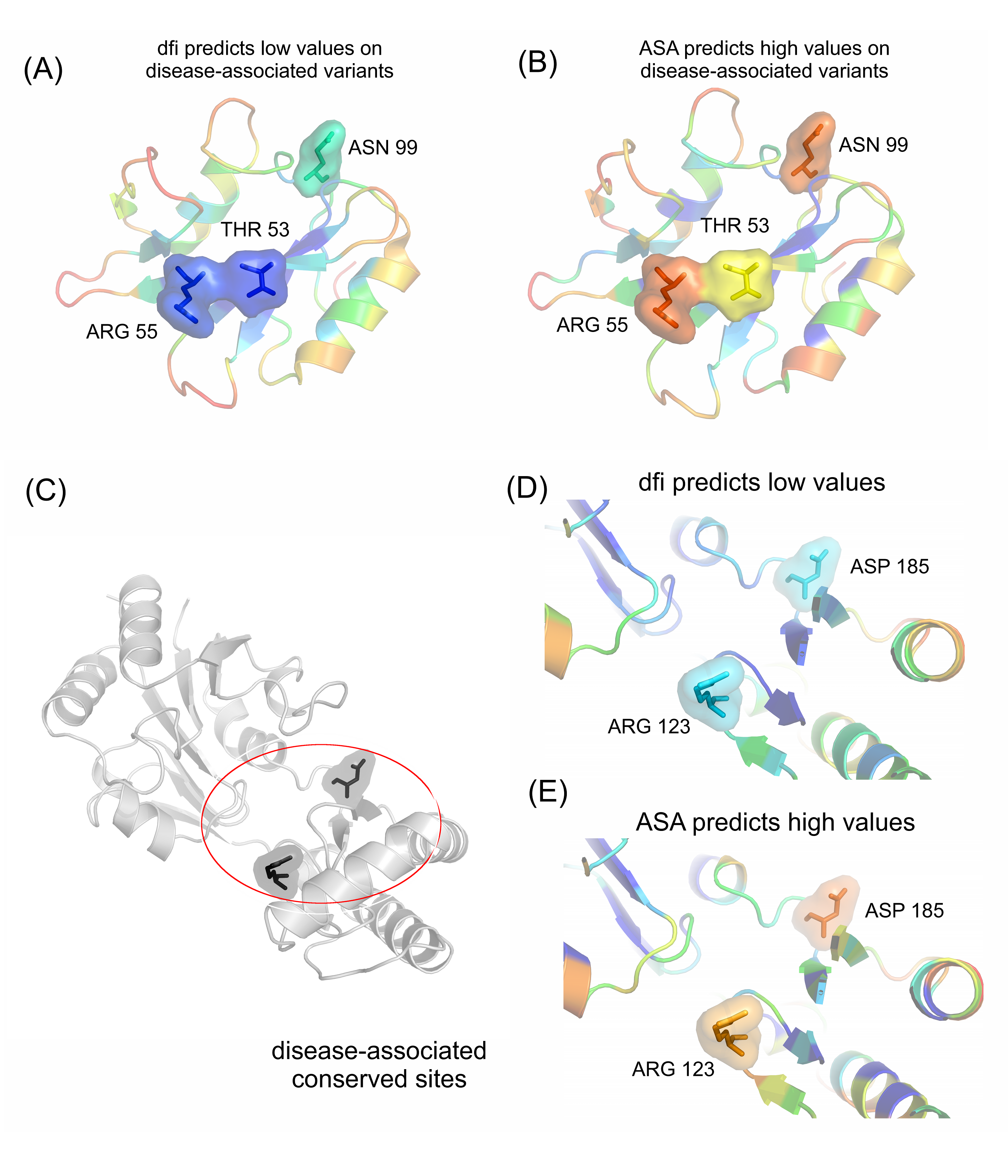


**Figure S2.** The color-coded ribbon diagrams of SH2 domain protein (NP_002342) with respect to (A) dynamic flexibility index, %*dfi* and (B) accessible surface area (%ASA) with in a spectrum of red-orange-yellow-cyan-blue, where red indicates highest values, and blue is the lowest value. These disease variants are shown in stick representations. Thr53 and Arg55 are binding sites. The average %*dfi* of these disease variants located in this protein is 15% whereas this value becomes 76% for average %ASA. The ribbon diagrams of human phosphomannomutase 2 (NP_000294) (C) of the whole chain (D) with color coded specific portion corresponding to the disease variants with respect to dynamic flexibility index, %*dfi* and (E) the same portion color coded with respect to accessible surface area, %ASA. The structure is colored within spectrum of red-yellow-green-cyan-blue where red shows the highest and blue the lowest values. Residues highlighted as surface representation are disease-associated variants. The average %*dfi* of these disease variants located in this protein is quite low 21% whereas this value becomes 78% for average %ASA.

**DataSet S1.** The list of proteins analyzed in this study. The protein accession codes are displayed in the first column. Their corresponding 3D-structures are obtained from the protein data bank (PDB) after applying a BLAST search. The dimeric or oligomeric proteins are treated as biological assemblies using the data provided by the experimentalists. Homologous proteins are also shown and labeled in the table. However, for all homologous sequences retrieved from the work of Kumar et al. 2009, we use a single structure with non-redundant set of positions that are involved in binding, catalytic activities or associated with neutral or disease alleles.

| NP_id | PDB id | Biological Assembly (Author provided in PDB) |
| --- | --- | --- |
| NP_000009 | 3B96 | Dimer |
| NP_000013 | 3IAR |  |
| NP_000020 | 2WXW^1^ |  |
| NP_000037 | 1FSU^2^ |  |
| NP_000068 | 1DC2 |  |
| NP_000087 | 2J5W |  |
| NP_000119 | 2F83^4^ | Dimer |
| NP_000210 | 2K21 |  |
| NP_000212 | 2HQQ | Dimer |
| NP_000282 | 2ZGV |  |
| NP_000286 | 1QLP |  |
| NP_000294 | 2AMY |  |
| NP_000300 | 3NKS |  |
| NP_000317 | 3HY5^3^ |  |
| NP_000342 | 1P49^2^ |  |
| NP_000361 | 1OIP^3^ |  |
| NP_000389 | 1UMK |  |
| NP_000420 | 2OBV | Dimer |
| NP_000593 | 1C5G^1^ |  |
| NP_000653 | 2PQT |  |
| NP_000666 | 2YDO |  |
| NP_000690 | 1B2Y |  |
| NP_000746 | 1NM8 |  |
| NP_000886 | 1HS6 |  |
| NP_000895 | 3FW1 | Dimer |
| NP_001045 | 1LS6 |  |
| NP_001070 | 2OZO |  |
| NP_001142 | 1OKC |  |
| NP_001227 | 2HRB |  |
| NP_001299 | 2NSM^5^ |  |
| NP_001376 | 2VR2 | Tetramer |
| NP_001436 | 1O1U |  |
| NP_001602 | 2BQ8 |  |
| NP_001614 | 2G2B |  |
| NP_001621 | 1W3W |  |
| NP_001701 | 2OK5^4^ |  |
| NP_001716 | 1EWF |  |
| NP_001748 | 1WMA |  |
| NP_001827 | 1NN6^4^ |  |
| NP_001877 | 3LWK | Dimer |
| NP_001899 | 1PBH |  |
| NP_002035 | 2A2D |  |
| NP_002044 | 1DG3 |  |
| NP_002141 | 3ISQ | Dimer |
| NP_002188 | 2B3X |  |
| NP_002328 | 2P01 |  |
| NP_002331 | 1W6J |  |
| NP_002334 | 1CB6 |  |
| NP_002342 | 1KA6 |  |
| NP_002442 | 1CB0 | Trimer |
| NP_002515 | 3CON |  |
| NP_002533 | 1KO9 |  |
| NP_002582 | 1KHB |  |
| NP_002601 | 2Q8F | Dimer |
| NP_002717 | 2XDW |  |
| NP_002745 | 3COI |  |
| NP_003271 | 1LA0 |  |
| NP_003332 | 3BZH | Dimer |
| NP_003346 | 2LCK |  |
| NP_003456 | 1WAW |  |
| NP_003928 | 3E9K | Dimer |
| NP_004047 | 2W2J |  |
| NP_004291 | 5PNT |  |
| NP_004521 | 1CK7 |  |
| NP_005031 | 3N2Z^5^ | Dimer |
| NP_005082 | 1YCK |  |
| NP_005201 | 2JDF^6^ |  |
| NP_005458 | 3FED | Dimer |
| NP_005501 | 3FQI |  |
| NP_005691 | 3FVY |  |
| NP_006220 | 2PPL^5^ |  |
| NP_006558 | 3CMQ |  |
| NP_006579 | 2AD1 |  |
| NP_006765 | 2A14 |  |
| NP_008822 | 1H4A^6^ |  |
| NP_009296 | 2PE4 |  |
| NP_036337 | 2QNK |  |
| NP_036407 | 1MD6 |  |
| NP_036519 | 3APM | Dimer |
| NP_037503 | 1N1F |  |
| NP_055177 | 3BPT |  |
| NP_055290 | 2O48 | Dimer |
| NP_057122 | 2KDO |  |
| NP_057240 | 1LW3 |  |
| NP_060293 | 2IMG |  |
| NP_060295 | 2FOZ |  |
| NP_064582 | 3TG4 |  |
| NP_064695 | 1ZUA |  |
| NP_612467 | 3I2N |  |
| NP_775293 | 2O9J |  |
| NP_001001438 | 1W6J |  |
| NP_001012982 | 1LO6^4^ |  |
| NP_001018119 | 3ACL |  |
| NP_001035181 | 3NKM |  |
| NP_001035998 | 2XDV | Dimer |
| NP_001073901 | 3LFM |  |
| NP_001094346 | 2OPW |  |
| NP_001127845 | 2JC9 |  |
| NP_001136401 | 2FY2 |  |
| NP_001137436 | 3QWL |  |

1. Serine protease inhibitor domain 2. Arylsulfatase domain 3. CRAL/Trio domain 4. Serine protease domain 5. Carboxypeptidase 6. Beta-crystallin S domain
